# Glycemic index, glycemic load, and metabolic syndrome in Mexican adolescents: a cross-sectional study from the NHNS-2012

**DOI:** 10.1186/s40795-017-0162-2

**Published:** 2017-05-25

**Authors:** Itandehui Castro-Quezada, Salomón Angulo-Estrada, Almudena Sánchez-Villegas, María Dolores Ruiz-López, Reyes Artacho, Lluís Serra-Majem, Teresa Shamah-Levy

**Affiliations:** 1 Research Institute of Biomedical and Health Sciences, University of Las Palmas de Gran Canaria, Luis Pasteur s/n, 35016 Las Palmas de Gran Canaria, Spain; 2 Department of Nutrition and Food Science, School of Pharmacy, University of Granada, Campus Universitario de la Cartuja, 18071 Granada, Spain; 3 Center for Nutrition and Health Research, National Institute of Public Health of Mexico, Universidad No. 655, Colonia Santa María Ahuacatitlán, 62100 Cuernavaca, Morelos Mexico; 4 Ciber Fisiopatología Obesidad y Nutrición (CIBEROBN, CB06/03), Instituto de Salud Carlos III (ISCIII), Spanish Government, Madrid, Spain; 5 Institute of Nutrition and Food Technologies, University of Granada, Avda. del Conocimiento, Armilla, 18100 Granada, Spain

**Keywords:** Glycemic index, Glycemic load, Metabolic syndrome, Adolescent, Mexico

## Abstract

**Background:**

The role of dietary glycemic index (GI) and dietary glycemic load (GL) on metabolic syndrome (MetS) in youth populations remains unclear. The aim of the present study was to evaluate the association among dietary GI, dietary GL, and MetS and its components in Mexican adolescents.

**Methods:**

This study was conducted within the framework of the National Health and Nutrition Survey 2012, a cross-sectional, probabilistic, population-based survey with a multistage stratified cluster sampling design. We analyzed a sample of 1346 subjects aged 12–19 years, representing 13,164,077 adolescents. Dietary habits were assessed through a validated semiquantitative food-frequency questionnaire. We assigned GI values using the International Tables of GI values. We defined MetS according to the International Diabetes Federation criteria developed for adolescents. Multiple logistic regression models were used to estimate odds ratios (ORs) and their 95% confidence intervals (CIs) to evaluate the association between categories of dietary GI and GL and the prevalence of MetS and its components.

**Results:**

We observed no associations between dietary GI or GL and MetS prevalence. Female adolescents in the highest category of dietary GI had higher odds of abnormal blood pressure (OR = 3.66; 95% CI, 1.46–9.22; *P* for trend = 0.012). A high dietary GL was also associated with higher odds of abnormal blood pressure in female adolescents (OR = 5.67; 95% CI, 1.84–17.46; *P* for trend = 0.003).

**Conclusions:**

We found higher odds of abnormal blood pressure for female adolescents with a high dietary GI and dietary GL.

## Background

The prevalence of metabolic syndrome (MetS) is high among children and adolescents with obesity [1, 2]. In Mexico, almost 35% of adolescents are either overweight or obese [3] and the prevalence of MetS oscillates between 6.5% [4] and 19.2% [5]. Therefore, special attention should be given to modifiable risk factors, such as lifestyle and dietary habits: they play an important role in the development and progression of MetS. Among dietary factors, carbohydrates are the main energy source in the diets of most populations and have a special function in energy metabolism and homoeostasis [6]. However, evidence indicates that some carbohydrate sources can be beneficial; others are not, depending on their quality and fiber content [7]. The quality of carbohydrates can be measured using the glycemic index (GI); this is defined as the incremental area under the curve of blood glucose response after eating 50 g of available carbohydrates from a certain food and expressed as a percentage of the glycemic response elicited by 50 g of glucose or white bread [8]. Moreover, the glycemic load (GL) considers both the quality and quantity of carbohydrate intake [9, 10].

In adults, evidence from different meta-analysis of randomized controlled trials (RCTs) demonstrated that low-GI or GL diets resulted in lower fasting blood glucose and glycated hemoglobin levels [11] and a greater decrease in total cholesterol and low density lipoprotein cholesterol (LDL-c) compared to control diets [12, 13]. Nevertheless, the latter findings have not been observed in overweight/obese subjects who followed low GI/GL diets [14]. Furthermore, results from RCTs have demonstrated a favorable effect of a low-GI diet on triglyceride levels [15] or concentration of high-density lipoprotein cholesterol (HDL-c) [16]. However, such findings are inconsistent and have not been confirmed by a recent meta-analysis [13].

In children and adolescents, a meta-analysis has demonstrated that low-GI diets might reduce serum triglycerides and homeostasis model assessment index in overweight or obese children and adolescents [17].

The association among GI, GL, and MetS has been mostly studied in prospective studies in adult populations [18, 19] and produced varying results. The evidence for such an association in young people is scarce. Two cross-sectional studies conducted in Australia have identified higher odds of developing MetS for each unit increase in breakfast GL [20] and per 20 unit dietary GL increase [21].

To our knowledge, no evidence is available on the relationship between the quality of carbohydrates and MetS in a Mexican youth population. Therefore, the main objective of this study was to evaluate the association among dietary GI, dietary GL, and MetS and its components in a nationally representative sample of Mexican adolescents.

## Methods

### Study population

This study was conducted within the framework of the National Health and Nutrition Survey 2012 (NHNS-2012), a cross-sectional, probabilistic, population-based survey with a multistage stratified cluster sampling design conducted in Mexico. The design and methods of the NHNS-2012 have been described elsewhere [22]. The main objective of the NHNS-2012 was to quantify the frequency, distribution, and trends in health and nutrition conditions and their determinants in the Mexican population [22]. Data were collected by computer-assisted interviews at participants’ homes. Child interviewees under the age of 14 years were assisted in their responses by a relative.

**Fig. 1:**
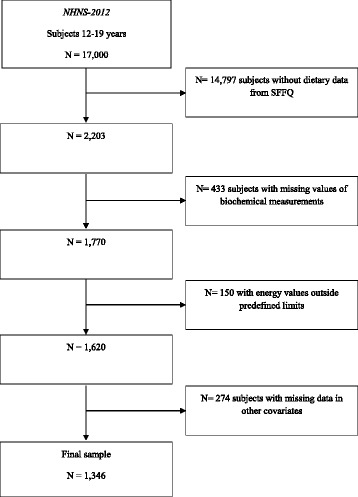
Flow chart showing study participant selection

In the NHNS-2012 an original probabilistic sample of 17,000 adolescents was drawn. For the present study, we used the NHNS-2012 subsample of 2203 adolescents aged 12–19 years evaluated by means of a validated semiquantitative food-frequency questionnaire (SFFQ) to assess dietary habits [23]. We excluded subjects with missing values for biochemical measurements (19.7%) or other covariates used in the statistical analyses (12.4%). Furthermore, we excluded subjects with energy values outside predefined limits (6.8%). The methodology for cleaning dietary data has been broadly described elsewhere [24]. First, the weight in grams of food consumed by each study subject was evaluated according to age-group. We excluded from the analysis subjects who consumed above three standard deviations (SDs) of one or more food items. The biological plausibility of food intake and the percentage contribution of each food to total dietary intake was used to verify data identified as high values. Second, we estimated very high values of energy intake by the ratio of energy intake/estimated energy requirement. The equations of the Institute of Medicine were used as reference [25]. The physical activity level of each subject was considered according to previous studies regarding data of the NHNS-1999 [26]. We excluded very low values of energy intake: under 0.5 of energy intake/basal metabolic rate (BMR). We estimated BMR for adults (≥19 years of age) using the Mifflin-St Jeor equations [27]. For subjects under 19 years of age, we used the age- and sex-specific equations of the Food and Agriculture Organization [28]. Accordingly, we included a final sample of 1346 subjects in our analyses, representing a total of 13,164,077 Mexican adolescents (Fig. 1).

### Exposure assessment

#### Dietary assessment

Trained personnel applied a validated SFFQ to evaluate dietary habits during the 7 days before the interview date [23, 24]. For each food item, the questionnaire measured the frequency of intake according to set categories: the range was “never” to “six times a day.” Participants also designated the food portion sizes, using defined categories and number of servings consumed during that week. We first converted the data to number of times a day, and we then estimated the daily portion size. To calculate the consumption of energy (kcal/day) and daily nutrient intakes, we multiplied the daily frequency of consumption (portions/day) of each food by the amount of energy and nutrients in a standard serving or portion size of that food. For that purpose, we used the food composition tables compiled by the National Institute of Public Health of Mexico (INSP: Databases of the nutritional value of food. Compilation of the National Institute of Public Health, unpublished). We totaled the contributions of all foods using Microsoft Visual FoxPro 7.0 (Microsoft Corporation, Seattle, WA, USA). The average Pearson correlation coefficient, between SFFQ and two 24-h dietary recalls, for absolute nutrient intake was 0.374 for adolescents. The unadjusted, adjusted and deattenuated Pearson correlation coefficients for carbohydrate intake in adolescent population were 0.51, 0.25 and 0.36 respectively [23]. The intake of carbohydrate, protein, fat, and dietary fiber was sex-specific adjusted for total energy intake using the residual method proposed by Willett et al. [29].

#### Dietary GI and dietary GL assessment

We used the protocol of Louie et al. [30] to assign a GI value to each food item in the SFFQ. We obtained the GI values from available studies conducted in normal subjects, using glucose as reference food [31, 32]. We calculated the dietary GI of each subject by summing the products of the available carbohydrate content per serving for each food multiplied by the average number of daily servings of that food multiplied by its GI; we then divided this by the total amount of daily carbohydrate intake [10, 33]. In a similar manner but without dividing by the total amount of carbohydrate, we estimated dietary GL [10]. Dietary GL was energy-adjusted using sex-specific residuals [29] owing to a high correlation with energy intake (*r* = 0.880, *P* < 0.001). Finally, we categorized dietary GI and energy-adjusted dietary GL into sex-specific tertiles.

### Outcome assessment

#### Anthropometric assessment

Weight and height were measured using electronic scales and wall stadiometers, respectively. We calculated the BMI as weight (kg) divided by height squared (m^2^). We used the BMI z-score (number of SDs by which a child differs from the mean BMI of children of the same age and sex) to classify subjects according to weight status as underweight, normal, overweight, or obese according to the World Health Organization (WHO) growth reference values for adolescents [34]. We measured waist circumference (WC) midway between the lowest rib and the iliac crest using an anthropometric tape parallel to the floor. Blood pressure was measured twice by a trained nurse in the dominant arm by means of a mercury sphygmomanometer [35]. The first reading was conducted after at least 5 min of seated rest. The second reading was taken 5 min after the first. The first Korotkoff sound was used as a measure for systolic blood pressure and the fifth sound for diastolic blood pressure.

#### Biochemical measurements

Fasting blood samples were collected by trained personnel of the NHNS-2012. The day before blood collection, subjects were instructed to avoid eating any solid or liquid food prior to collection. Blood was drawn from an antecubital vein and collected in tubes without anticoagulant. The blood was centrifuged in situ at 3000 g. For subjects who reported a previous diagnosis of type 2 diabetes mellitus (T2D), a second sample was collected in heparinized tubes. Serum aliquots were stored in cryovials and frozen in liquid nitrogen. Samples were transported to the Mexican National Institute of Public Health and stored at −70 °C for posterior analyses in the biochemistry laboratory.

We measured serum glucose concentrations using the glucose oxidase method through chemiluminescence with an automated analyzer (Architect ci8200, Abbott Diagnostics, Wiesbaden, Germany). To verify the accuracy and precision of the procedure, the 965 material of the National Institute of Standards and Technology was measured simultaneously. We determined serum triglyceride levels after lipase hydrolysis in an automatic analyzer (Architect ci8200, Abbott Diagnostics, Wiesbaden, Germany). HDL-c was measured using an enzymatic colorimetric direct method after eliminating chylomicrons, very-low-density lipoproteins (VLDL), and low-density lipoproteins by enzymatic digestion. To assure the precision and accuracy of these measurements, the concentrations of HDL-c and triglycerides were measured simultaneously at a second laboratory (Lipids Laboratory, National Institute of Medical Science and Nutrition Salvador Zubiran of Mexico).

#### Metabolic syndrome

The presence of MetS was identified according to the International Diabetes Federation (IDF) definition of MetS for children and adolescents [36, 37]. For adolescents aged 12–16 years, MetS was defined according to the following criteria: (1) presence of abdominal obesity (WC ≥90th percentile for age and sex or adult cutoff if lower); and (2) the presence of two or more other conditions among triglycerides ≥150 mg/dL, HDL-c <40 mg/dL, systolic blood pressure ≥130 or diastolic blood pressure ≥85 mmHg, fasting plasma glucose ≥100 mg/dL, and known T2D. Adult IDF criteria were used for subjects aged 16 years or older: central obesity (defined as WC ≥90 cm for male and ≥80 cm for female adolescents); and at least two of the following factors: triglycerides ≥150 mg/dL or specific treatment for high triglycerides; HDL-c <40 mg/dL in males and <50 mg/dL in females or specific treatment for these lipid abnormalities; systolic blood pressure ≥130, diastolic blood pressure ≥85 mmHg, or treatment of previously diagnosed hypertension; fasting plasma glucose ≥100 mg/dL; or previously diagnosed T2D.

### Covariates

We used specific questionnaires to assess sociodemographic characteristics, medical history, and lifestyle habits. Socioeconomic status (SES) information was based on well-being. Using these data, we calculated an index (well-being index) by principal-components analysis, which included home conditions and presence in the home of household appliances, goods, and services. The continuous variable was categorized into tertiles and used as a proxy for low, medium, and high SES levels.

To collect information on physical activity and sedentary lifestyle in the 12- to 14-year age-group, we used a questionnaire of eight items [38]. The questions included hours of sleep, screen time, means of transportation to school, and formal physical activity (e.g., skating, dancing, and soccer) over the previous year. We also identified the means of transportation and length of time spent on the home-to-school route and vice versa. Furthermore, we categorized formal or competitive physical activities performed in the previous year according to the following criteria: (1) inactive; (2) one or two activities; and (3) three or more activities.

We assessed physical activity in adolescents aged 15–19 years using the short version of the International Physical Activity Questionnaire [39]. In addition, participants were asked about their usual hours of sleep, inactive transport time, and usual screen time [40, 41]. The evaluation comprised 14 questions and allowed us to differentiate the activity during the week and on weekends. Finally, in agreement with WHO criteria, we classified physical activity into three categories: active, moderately active, and inactive [42].

### Statistical analyses

The sample design characteristics (sample weights, cluster, and strata variables) were considered for all the analyses. We estimated the baseline characteristics of the population and dietary intake according to sex-specific tertiles of dietary GI and energy-adjusted dietary GL. To explore differences across categories of dietary GI and energy-adjusted dietary GL, we used linear regression models and design-based Wald statistics for quantitative variables; we employed the design-based F statistic (corrected, weighted Pearson chi-square statistic) for categorical data.

We used multiple logistic regression models to estimate odds ratios (ORs) and their 95% confidence intervals (CIs) to evaluate the association between categories of dietary GI and GL and the prevalence of MetS. The first model was adjusted for age (years). The second multivariate model further included the following: SES (low, middle, high); geographic regions of Mexico (north, central, south, metropolitan area) and dietary fiber intake (continuous, energy-adjusted). To examine the associations between categories of dietary GI and GL and the prevalence of MetS components (elevated WC, abnormal blood pressure, elevated fasting serum triglycerides, low HDL-c, elevated fasting serum glucose concentrations), we fitted logistic regression models with the same covariates as those used for the main analyses. We selected covariates using a hypothesis-based analysis. The addition of potential confounders, such as physical activity levels or screen time as covariates in the multivariate models, did not change the magnitude or effect of our results; thus, we did not use those factors in the final models. We took the lowest categories of dietary GI and GL as references in all the models. The tests of the linear trend across increasing categories of dietary GI and GL were conducted by assigning the sex-specific median value within each category. We treated those variables as continuous in the logistic regression models.

To examine a possible interaction between dietary GI and GL and age (under and over 16 years), and weight status (underweight/normal, overweight/obese), we introduced the product terms in the different multivariable models; we considered *P* < 0.05 in the likelihood ratio test as statistically significant. All statistical analyses were performed using Stata 12.0 (StataCorp, College Station, TX, USA), and the significance level was set at *P* < 0.05.

## Results

In this study, the mean (SD) dietary GI and GL of adolescents in the NHNS-2012 was 51.8 (5.3) and 150.0 (27.3), respectively. The MetS prevalence in the overall sample was 8.8%, with a higher proportion among female (12.0%) than male adolescents (6.4%; *P* = 0.019).

Tables 1 and 2 present the main characteristics of the sample according to sex-specific tertiles of dietary GI and energy-adjusted dietary GL. Participants in the highest category of dietary GI had higher carbohydrate and sugar intake and lower values of protein and total fat, than subjects in the lowest category of dietary GI. Similar characteristics were found across categories of dietary GL, in addition, we observed a higher dietary fiber intake in the top tertile of dietary GL compared with those in the lowest tertile. We found no differences in the prevalence of MetS or the mean of its components across dietary GL categories.

**Table 1:** General characteristics of the sample according to sex-specific categories of dietary glycemic index^a^

*Characteristics*	*Dietary glycemic index* ^b^													
	*Female adolescents*							*Male adolescents*						
	Low		Moderate		High		*P value*	Low		Moderate		High		*P value*
	Mean	SD	Mean	SD	Mean	SD		Mean	SD	Mean	SD	Mean	SD	
Dietary GI (units)	46.8	0.3	51.0	0.1	56.6	0.4	<0.001	47.6	0.3	51.9	0.1	57.0	0.3	<0.001
Age (years)	15.5	0.2	16.2	0.3	16.0	0.2	0.123	15.4	0.2	16.0	0.2	15.6	0.2	0.185
Socioeconomic status (%)							0.549							0.117
Low	30.0		25.6		30.9			27.7		32.6		31.2		
Medium	26.8		34.3		35.6			37.3		28.1		35.9		
High	43.3		40.1		33.5			35.0		39.3		32.9		
Geographic region (%)							0.186							0.515
North	24.4		12.2		20.8			19.5		14.4		25.0		
Central	25.1		25.2		35.4			31.0		28.9		31.6		
Metropolitan area	24.5		30.3		15.4			14.3		19.8		12.0		
South	26.1		32.3		28.4			35.2		37.0		31.4		
Weight status (%)							0.379							0.001
Underweight	1.3		1.0		1.9			1.2		0.9		0.7		
Normal	64.2		65.9		50.9			71.8		66.3		69.7		
Overweight	21.3		23.4		34.5			13.1		28.0		13.3		
Obese	13.3		9.7		12.7			13.9		4.9		16.3		
Screen time (computer, TV, and video) (%)							0.131							0.011
≤ 2 h/day	29.6		43.8		28.9			33.7		39.8		29.4		
2–4 h/day	35.0		29.1		45.7			29.3		39.5		37.1		
≥ 4 h/day	34.1		25.3		24.5			36.8		17.8		33.5		
No data available	13		1.7		1.0			0.3		2.9		0.0		
Physical activity (%, age 12–14 years)							0.174							0.034
Sedentary	76.3		77.7		57.7			51.6		40.2		55.9		
1–2 activities	18.9		20.0		36.4			44.9		50.5		41.1		
≥ 3 activities	2.1		0.5		2.4			2.7		0.6		2.5		
No data available	2.7		1.8		3.4			0.7		8.6		0.5		
Physical activity (%, age 15–19 years)							0.239							0.131
Sedentary	28.2		29.7		26.7			17.7		17.9		14.0		
Moderately active	6.1		22.2		16.7			9.4		19.8		19.4		
Active	65.8		46.0		56.7			72.9		62.3		66.6		
No data available	0.0		2.1		0.0			0.0		0.0		0.0		
Dietary intake														
Total energy intake (kcal/d)	1795	53	1747	73	1828	58	0.707	2033	53	2121	61	2215	69	0.118
Carbohydrate intake (g/d)^c^	246.0	3.3	271.5	4.2	269.7	3.3	<0.001	298.0	3.6	315.8	4.3	311.5	4.1	0.002
Carbohydrate intake (% energy)	54.9	0.7	61.5	1.2	60.8	0.8	<0.001	55.8	0.7	59.5	0.8	59.1	0.7	<0.001
Protein intake (g/d)^c^	58.5	1.0	52.7	1.2	51.9	0.8	<0.001	67.9	1.1	63.8	1.0	60.5	1.2	<0.001
Protein intake (% energy)	13.2	0.2	11.8	0.3	11.5	0.2	<0.001	13.0	0.2	12.1	0.2	11.4	0.2	<0.001
Fat intake (g/d)^c^	67.2	1.1	58.8	1.3	59.1	1.3	<0.001	77.5	1.3	70.5	1.6	69.5	1.5	<0.001
Fat intake (% energy)	33.9	0.5	28.8	0.9	29.4	0.7	<0.001	33.2	0.5	29.8	0.7	29.6	0.5	<0.001
MUFA (g/d)^c^	22.6	0.5	19.7	0.5	20.5	0.6	<0.001	25.9	0.6	23.6	0.5	24.7	0.6	0.020
PUFA (g/d)^c^	14.3	0.4	14.1	0.4	14.5	0.4	0.750	17.4	0.4	17.5	0.7	16.8	0.4	0.424
SFA (g/d)^c^	25.8	0.5	22.1	0.6	22.7	0.7	<0.001	29.4	0.7	26.2	0.7	26.5	0.7	0.002
Trans fatty acids (g/d)^c^	0.5	0.0	0.5	0.0	0.5	0.0	0.054	0.5	0.0	0.5	0.0	0.6	0.0	0.692
Dietary fiber intake (g/d)^c^	21.6	0.5	22.7	1.3	21.3	0.7	0.683	25.8	0.7	27.0	1.0	22.8	0.8	0.003
Dietary sugar intake (g/d)	94.1	4.9	109.9	7.2	112.3	4.4	0.021	108.4	4.1	116.2	6.4	134.6	4.4	<0.001
WC (cm)	76.8	1.0	76.8	1.2	78.3	1.4	0.640	77.0	1.5	77.5	1.0	78.7	1.2	0.660
Triglycerides (mg/dL)	116.9	7.5	135.3	9.7	113.5	5.6	0.142	113.2	6.6	113.3	6.6	132.1	9.4	0.212
HDL-c (mg/dL)	45.1	0.8	48.7	2.0	43.0	1.4	0.075	43.3	0.9	41.3	0.9	43.0	0.8	0.231
Systolic blood pressure (mmHg)	107.1	0.9	108.9	1.1	110.0	1.2	0.169	111.2	1.5	110.9	1.0	113.3	1.0	0.219
Diastolic blood pressure (mmHg)	70.0	0.8	72.0	1.1	73.2	1.0	0.050	70.3	1.1	71.1	0.9	73.3	0.8	0.051
Fasting serum glucose (mg/dL)	80.4	1.0	79.0	1.2	77.6	1.0	0.172	81.3	0.8	80.2	1.3	81.5	1.4	0.733
MetS prevalence (%)^d^	9.5		9.7		16.9		0.234	6.9		3.8		8.4		0.344

*Abbreviations: GI* Glycemic index, *GL* glycemic load, *kcal/d* kilocalories per day, grams per day (g/d), *MUFA* monounsaturated fatty acids, *PUFA* polyunsaturated fatty acids, *SFA* saturated fatty acids, *WC* waist circumference, *HDL-c* high-density lipoprotein cholesterol, *MetS* metabolic syndrome

^a^Values are expressed as means and standard deviations (SD) for continuous variables, and data from categorical variables are shown as percentages

^b^Categories based on sex-specific tertiles of dietary GI. ^c^Values were adjusted for energy intake using sex-specific residuals. ^d^The age-specific International Diabetes Foundation definition of the metabolic syndrome was used [36, 37]

**Table 2:** General characteristics of the sample according to sex-specific categories of energy-adjusted dietary glycemic load^a^

*Characteristics*	*Energy-adjusted dietary glycemic load* ^bc^													
	*Female adolescents*							*Male adolescents*						
	Low		Moderate		High		*P value*	Low		Moderate		High		*P value*
	Mean	SD	Mean	SD	Mean	SD		Mean	SD	Mean	SD	Mean	SD	
Dietary GL^c^ (units)	110.0	1.3	135.0	0.6	162.9	1.4	<0.001	132.1	1.5	159.9	0.7	192.5	1.4	<0.001
Age (years)	15.9	0.3	15.9	0.2	15.9	0.3	0.998	15.8	0.3	15.9	0.2	15.3	0.2	0.112
Socioeconomic status (%)							0.013							<0.001
Low	21.3		30.6		34.7			16.9		29.9		44.8		
Medium	24.9		40.4		31.4			31.3		33.2		36.7		
High	53.8		29.0		33.9			51.8		37.0		18.5		
Geographic region (%)							0.030							0.065
North	26.8		19.3		11.3			23.2		22.7		12.9		
Central	29.6		26.7		29.4			26.2		29.4		35.9		
Metropolitan area	28.1		15.6		26.5			23.1		12.5		10.6		
South	15.6		38.4		32.8			27.6		35.4		40.7		
Weight status (%)							0.678							0.283
Underweight	1.3		1.8		1.2			0.7		0.9		1.1		
Normal	63.7		55.0		62.3			72.1		61.5		74.1		
Overweight	20.4		31.1		27.6			15.0		24.3		15.3		
Obese	14.6		12.1		9.0			12.2		13.3		9.5		
Screen time (computer, TV, and video) (%)							0.868							<0.001
≤ 2 h/day	35.8		32.8		33.7			35.9		27.6		39.6		
2–4 h/day	32.7		37.1		40.1			23.5		47.9		34.6		
≥ 4 h/day	31.2		28.1		24.6			40.3		24.3		23.2		
No data available	0.4		2.0		1.6			0.3		0.3		2.6		
Physical activity (%, age 12–14 years)							0.039							0.171
Sedentary	79.3		69.3		61.7			56.4		50.5		44.0		
1–2 activities	16.6		24.3		35.6			42.3		46.6		46.5		
≥ 3 activities	3.2		0.0		2.2			0.6		2.0		3.2		
No data available	1.0		6.4		0.5			0.7		0.9		6.3		
Physical activity (%, age 15–19 years)							0.255							0.355
Sedentary	37.7		26.3		21.2			14.1		20.4		15.1		
Moderately active	11.5		12.2		22.9			8.0		23.0		19.3		
Active	50.8		61.5		53.7			77.9		56.6		65.7		
No data available	0.0		0.0		2.3			0.0		0.0		0.0		
Dietary intake														
Total energy intake (kcal/d)	1844	58	1697	59	1828	62	0.190	2169	53	2019	65	2179	60	0.141
Carbohydrate intake (g/d)^c^	228.2	2.1	263.0	1.8	296.5	2.3	<0.001	266.1	2.8	308.6	2.0	350.9	2.9	<0.001
Carbohydrate intake (% energy)	50.8	0.5	59.5	0.4	67.0	0.7	<0.001	50.2	0.5	58.2	0.4	66.1	0.5	<0.001
Protein intake (g/d)^c^	61.2	0.8	53.4	0.7	48.5	1.0	<0.001	71.4	1.2	64.1	0.8	56.8	0.9	<0.001
Protein intake (% energy)	13.8	0.2	12.0	0.2	10.8	0.2	<0.001	13.6	0.2	12.2	0.2	10.7	0.2	<0.001
Fat intake (g/d)^c^	73.0	0.9	61.9	0.8	50.1	0.9	<0.001	85.6	1.2	72.8	0.7	59.1	1.1	<0.001
Fat intake (% energy)	36.9	0.4	30.6	0.4	24.6	0.6	<0.001	36.5	0.5	30.9	0.4	25.1	0.4	<0.001
MUFA (g/d)^c^	25.0	0.5	21.0	0.4	16.8	0.4	<0.001	28.8	0.6	25.1	0.3	20.2	0.5	<0.001
PUFA (g/d)^c^	14.9	0.4	14.3	0.4	13.8	0.4	0.130	19.0	0.7	16.6	0.3	16.1	0.4	0.002
SFA (g/d)^c^	28.6	0.6	23.7	0.4	18.3	0.4	<0.001	32.8	0.6	28.0	0.4	21.3	0.6	<0.001
Trans fatty acids (g/d)^c^	0.6	0.0	0.5	0.0	0.4	0.0	<0.001	0.7	0.0	0.5	0.0	0.4	0.0	<0.001
Dietary fiber intake (g/d)^c^	18.7	0.7	21.0	0.5	25.9	1.1	<0.001	20.9	0.5	24.6	0.7	30.2	0.9	<0.001
Dietary sugar intake (g/d)	94.4	6.0	102.5	5.5	119.6	6.2	0.014	111.7	3.9	117.3	5.6	129.9	5.2	0.015
WC (cm)	77.5	1.2	78.1	1.1	76.3	1.4	0.592	78.1	1.5	79.1	1.3	76.0	1.1	0.133
Triglycerides (mg/dL)	117.5	6.6	115.0	8.2	133.3	9.4	0.300	114.0	7.7	121.5	6.9	122.8	8.4	0.711
HDL-c (mg/dL)	47.8	2.0	44.1	1.0	44.9	1.5	0.233	43.0	0.9	41.6	0.8	43.1	0.9	0.302
Systolic blood pressure (mmHg)	108.2	0.9	108.6	1.0	109.3	1.3	0.794	111.5	1.6	112.0	0.9	111.8	0.9	0.955
Diastolic blood pressure (mmHg)	71.3	0.9	71.3	1.0	72.5	1.1	0.653	70.5	1.1	71.5	0.9	72.7	0.8	0.231
Fasting serum glucose (mg/dL)	80.4	1.2	79.9	1.2	76.7	0.9	0.018	81.4	1.0	81.5	1.4	80.1	1.2	0.620
MetS prevalence (%)^d^	9.1		13.0		13.9		0.605	8.7		5.2		5.2		0.461

*Abbreviations: GI* Glycemic index, *GL* glycemic load, *kcal/d* kilocalories per day, *g/d* grams per day, *MUFA* monounsaturated fatty acids, *PUFA* polyunsaturated fatty acids, *SFA* saturated fatty acids, *WC* waist circumference, *HDL-c* high-density lipoprotein cholesterol, *MetS* metabolic syndrome

^a^Values are expressed as means and standard deviations (SD) for continuous variables, and data from categorical variables are shown as percentages

^b^Categories based on sex-specific tertiles of dietary GL. ^c^Values were adjusted for energy intake using sex-specific residuals. ^d^The age-specific International Diabetes Foundation definition of the metabolic syndrome was used [36, 37]

Table 3 shows the ORs and 95% CI for MetS and its components according to sex-specific categories of dietary GI. We observed no association of MetS with either dietary GI or dietary GL. However, when MetS components were analyzed separately, a direct association between the highest dietary GI and abnormal blood pressure was evident in female adolescents (Model 1: OR = 3.66; 95% CI, 1.59–8.39; *P* for trend = 0.009). This association remained statistically significant after multivariate adjustment. Table 4 shows the ORs and 95% CI for MetS and its components according to sex-specific categories of energy-adjusted dietary GL. Our results from the multivariate model also indicated that female adolescents with the highest dietary GL had higher odds of abnormal blood pressure (OR = 5.67; 95% CI, 1.84–17.46); there was a significant trend across categories of dietary GL (*P* for trend = 0.003). Among males, no statistically significant associations were found between dietary GI or dietary GL and abnormal BP. We found no statistically significant associations for the remaining MetS criteria with dietary GI or GL.

**Table 3:** Association between metabolic syndrome and sex-specific categories of dietary glycemic index

	*Dietary glycemic index* ^a^							
	*Female adolescents*				*Male adolescents*			
	Low	Moderate	High	*P* trend	Low	Moderate	High	*P* trend
MetS								
Model 1^b^ OR (95% CI)	1	0.92 (0.35–2.40)	1.78 (0.70–4.55)	0.192	1	0.50 (0.15–1.63)	1.21 (0.50–3.19)	0.673
Model 2^c^ OR (95% CI)	1	0.81 (0.30–2.19)	1.60 (0.62–4.15)	0.275	1	0.56 (0.18–1.77)	1.25 (0.48–3.31)	0.641
Elevated WC								
Model 1^b^ OR (95% CI)	1	1.22 (0.58–2.54)	1.24 (0.70–2.22)	0.486	1	0.84 (0.40–1.74)	1.13 (0.58–2.20)	0.696
Model 2^c^ OR (95% CI)	1	1.16 (0.56–2.42)	1.33 (0.72–2.45)	0.361	1	0.87 (0.43–1.76)	1.25 (0.64–2.46)	0.513
Elevated triglycerides								
Model 1^b^ OR (95% CI)	1	1.41 (0.64–3.12)	0.98 (0.51–1.88)	0.840	1	0.80 (0.38–1.66)	1.06 (0.53–2.12)	0.850
Model 2^c^ OR (95% CI)	1	1.25 (0.58–2.68)	0.99 (0.52–1.88)	0.911	1	0.78 (0.35–1.70)	1.15 (0.56–2.35)	0.714
Low HDL-c								
Model 1^b^ OR (95% CI)	1	0.69 (0.37–1.29)	1.65 (0.93–2.94)	0.058	1	1.58 (0.91–2.76)	1.26 (0.76–2.09)	0.396
Model 2^c^ OR (95% CI)	1	0.67 (0.36–1.26)	1.56 (0.82–2.95)	0.126	1	1.71 (1.00–2.92)	1.29 (0.78–2.13)	0.321
Abnormal blood pressure								
Model 1^b^ OR (95% CI)	1	2.22 (0.76–6.43)	3.66 (1.59–8.39)	0.009	1	0.48 (0.18–1.28)	1.67 (0.82–3.40)	0.139
Model 2^c^ OR (95% CI)	1	2.02 (0.60–6.75)	3.66 (1.46–9.22)	0.012	1	0.53 (0.20–1.41)	1.66 (0.83–3.32)	0.143
Elevated fasting serum glucose								
Model 1^b^ OR (95% CI)	1	0.71 (0.13–3.76)	0.24 (0.05–1.32)	0.114	1	1.25 (0.23–6.63)	2.30 (0.47–11.22)	0.278
Model 2^c^ OR (95% CI)	1	1.07 (0.23–5.11)	0.24 (0.05–1.22)	0.068	1	1.21 (0.24–6.17)	2.72 (0.43–17.08)	0.289

*Abbreviations: OR* Odds ratio, *CI* confidence interval, *MetS* metabolic syndrome, *WC* waist circumference, *HDL-c* high-density lipoprotein cholesterol. ^a^Categories based on sex-specific tertiles of dietary GI. ^b^Model adjusted for age (years). ^c^Multivariate model adjusted for age (years), socioeconomic level (low, middle, or high), geographic region (north, central, south, or metropolitan area) and dietary fiber intake (continuous, energy-adjusted)

**Table 4:** Association between metabolic syndrome and sex-specific categories of energy-adjusted dietary glycemic load

	*Energy-adjusted dietary glycemic load* ^ab^							
	*Female adolescents*				*Male adolescents*			
	Low	Moderate	High	*P* trend	Low	Moderate	High	*P* trend
MetS								
Model 1^c^ OR (95% CI)	1	1.52 (0.56–4.12)	1.64 (0.60–4.49)	0.338	1	0.57 (0.19–1.68)	0.59 (0.20–1.75)	0.364
Model 2^d^ OR (95% CI)	1	1.48 (0.54–4.04)	1.88 (0.64–5.55)	0.255	1	0.50 (0.15–1.64)	0.55 (0.18–1.67)	0.310
Elevated WC								
Model 1^c^ OR (95% CI)	1	1.19 (0.63–2.25)	1.12 (0.55–2.29)	0.760	1	0.85 (0.40–1.82)	0.93 (0.46–1.89)	0.863
Model 2^d^ OR (95% CI)	1	1.23 (0.65–2.36)	1.07 (0.52–2.20)	0.848	1	0.85 (0.39–1.84)	0.95 (0.45–2.00)	0.906
Elevated triglycerides								
Model 1^c^ OR (95% CI)	1	0.93 (0.45–1.96)	1.61 (0.72–3.59)	0.240	1	1.11 (0.54–2.27)	1.03 (0.50–2.11)	0.954
Model 2^d^ OR (95% CI)	1	0.80 (0.38–1.71)	1.03 (0.46–2.29)	0.909	1	0.92 (0.43–1.94)	0.67 (0.30–1.48)	0.300
Low HDL-c								
Model 1^c^ OR (95% CI)	1	1.80 (0.96–3.40)	1.93 (0.96–3.88)	0.074	1	1.31 (0.76–2.25)	0.94 (0.54–1.64)	0.773
Model 2^d^ OR (95% CI)	1	1.44 (0.78–2.67)	1.73 (0.82–3.64)	0.151	1	1.13 (0.65–1.97)	0.76 (0.41–1.41)	0.351
Abnormal blood pressure								
Model 1^c^ OR (95% CI)	1	1.07 (0.36–3.23)	2.69 (0.93–7.78)	0.073	1	0.66 (0.30–1.42)	1.00 (0.47–2.12)	0.948
Model 2^d^ OR (95% CI)	1	1.42 (0.42–4.79)	5.67 (1.84–17.46)	0.003	1	0.69 (0.32–1.48)	1.30 (0.56–3.03)	0.538
Elevated fasting serum glucose								
Model 1^c^ OR (95% CI)	1	0.52 (0.13–2.16)	0.42 (0.05–3.68)	0.416	1	1.94 (0.42–8.99)	2.20 (0.46–10.42)	0.313
Model 2^d^ OR (95% CI)	1	0.52 (0.13–2.11)	0.62 (0.10–3.83)	0.568	1	2.35 (0.45–12.24)	3.43 (0.38–30.80)	0.260

*Abbreviations: OR* Odds ratio, *CI* confidence interval, *MetS* metabolic syndrome, *WC* waist circumference, *HDL-c* high-density lipoprotein cholesterol. ^a^Categories based on sex-specific tertiles of dietary GL. ^b^Values were adjusted for energy intake using sex-specific residuals. ^c^Model adjusted for age (years). ^d^Multivariate model adjusted for age (years), socioeconomic level (low, middle, or high), geographic region (north, central, south, or metropolitan area) and dietary fiber intake (continuous, energy-adjusted).

None of the interactions assessed was statistically significant in the association between dietary GI and GL and MetS (*P* for interaction >0.05)

## Discussion

In this cross-sectional study, we found no associations between dietary GI or GL and MetS. However, in an analysis of MetS components, high dietary GI and GL were associated with higher odds of abnormal blood pressure in female adolescents.

We found no associations between dietary GI or GL and MetS. Similar results were observed in a clinical trial performed in European children and adolescents (5–18 years) did not reveal an association between a low-GI diet and MetS [43]. A cross-sectional study conducted in 516 Australian adolescents found no association between overall dietary GI or dietary GL and MetS [20]. In that study, however, breakfast GL was found to be predictive of MetS in female, but not male, adolescents. In the present study, we used SFFQ to assess dietary intake, and we were unable to estimate dietary GI or GL at different mealtimes. Thus, it was not possible for us to confirm the results of that Australian study.

Our results also contrast with those of a cross-sectional study, in which dietary GL was associated with a higher prevalence of MetS in 769 adolescents (13–15 years) [21]. The variance with our results may be explained by the different methods used for dietary assessment. The 3-day food record used in that study may in fact have assessed GI more accurately than the SFFQ used in ours: food records give a more precise indication of the types and portions of food consumed than the SFFQ.

We identified an association between the highest dietary GI and GL and abnormal blood pressure among female adolescents. In contrast to our findings, those of a clinical trial that included 50 overweight or obese female adolescents did not indicate a decrease in blood pressure after a 10-weeks intervention with a low-GI diet [44]. The discrepancy between our results and theirs could be explained by the study design. Our cross-sectional study did not allow an assessment of causality; therefore, more prospective studies and clinical trials are needed to confirm the observed association. On the other hand, similar results were observed in a prospective investigation conducted among 858 Australian adolescents followed up for 5 years [45]. The authors found a direct association among female adolescents: for each 1-SD increment in dietary GI and GL, mean systolic blood pressure rose by 2.3 and 4 mmHg, respectively. In that study, no significant associations were observed between carbohydrate quality and blood pressure among male adolescents.

In the present work, no evidence was found concerning an association among dietary GI, dietary GL, and the remaining METs components (elevated WC, elevated triglycerides, low HDL-c, elevated fasting serum glucose). Results from a recent systematic review did not show an association between low/high GI diets and body mass index, waist circumference, hip circumference, waist-to-hip ratio, total cholesterol, LDL-c, HDL-c, diastolic and systolic blood pressure, fasting serum glucose, fasting serum insulin, glycosylated hemoglobin and C-reactive protein. However, the latter meta-analyses demonstrated that low GI protocols resulted in more pronounced decreases in triglycerides and HOMA-index [17].

Nevertheless, recent intervention studies determined that low-GI diets led to a significantly greater reduction in WC [46, 47] compared with controls. Also, a clinical trial have demonstrated that blood glucose total area under the curve was 13% greater with a high-GI than low-GI breakfast among overweight female adolescents and 4% higher in non-overweight female adolescents [48]. Moreover, a dietary intervention with low GI was observed to improve serum glucose levels in children and adolescents with type 1 diabetes mellitus [49]. Similarly, a low-GL dietary intervention for 6 weeks among overweight and obese 11-year-old children showed a reduction in fasting glucose [50]. However, clinical trials have been conducted in specific population groups: this fact—along with dietary intervention—could explain the differences from our results.

In our study, mean dietary GI was 51.5 among female adolescents and 52.1 in male adolescents, and dietary GL was higher among male adolescents (161.4) than female adolescents (135.8). The GI values of our sample were lower than those found in Australian, Canadian, British or Japanese adolescents (around 56 to 64 units) and mean dietary GL of our study was in agreement to previous studies conducted in adolescents (range from 128 to 168 units) [20, 51–54]. Thus it is still necessary and urgent to elucidate the role that low GI or GL diets exert on MetS onset in youth population worldwide, since individuals with MetS have a 2-fold risk of developing cardiovascular disease [55] and higher risk of T2D compared with people without this syndrome [56].

One hypothesized metabolic effect by which high-GI and GL diets increase blood pressure is a postprandial glycemic response and the consequent hyperinsulinemia elicited after consuming high-GI foods [57]. It has been found that higher dietary GI during puberty is prospectively associated with greater insulin resistance [58]. Hyperinsulinemia has been associated with abnormal levels of blood pressure through stimulation of the sympathetic nervous system [59], increased sodium retention, and volume expansion [45].

We acknowledge that our study has several limitations. Owing to the cross-sectional design, we cannot make causal inferences. Our findings are specific for Mexican adolescents and cannot therefore be generalized to other population groups. Other limitation is that we were unable to assess the impact of pubertal or hormonal status in our analyses. Puberty could be a confounding variable since transition from Tanner stage I to Tanner stage III has been associated with temporary reduction of insulin sensitivity, increases in fasting glucose and insulin levels and different hormonal changes [60]. In addition, physical activity was not included as a covariate in our analyses due to the lack of significance in our models. However, a recent meta-analysis has found an association between physical activity and MetS in adolescents [61]. We therefore, cannot discard that measurement error might exist since questionnaires used in this study are not validated for estimating physical activity in Mexican adolescents. Also, underreporting could be a source of bias in our study, since evidence in adolescents demonstrated that misreporters showed higher rates of insufficient intake of carbohydrate [62]. Although in our study subjects with energy values outside predefined limits were excluded, under-reporting bias might still exist and alter the estimation of nutrient intake and the associations between dietary GI or GL and MetS.

Moreover, the SFFQ evaluated consumption of foods during 7 days prior to the date of the interview, thus habitual dietary habits of the population might not be reflected by this assessment. In addition, the SFFQ was not specifically designed to evaluate dietary GI and GL; using this tool could generate bias about dietary GI and GL variation owing to the limited number of food items and restrictions in quantifying individual amounts of food consumed [63]. Nevertheless, the SFFQ used in the NHNS-2012 has been found sufficiently valid for assessing carbohydrate intake in adolescents [23]. Furthermore, published GI values for local foods in Mexico are limited; for that reason, we used reference GI data from other countries. This could be a source of error because GI values of foods may differ according to variety, growing conditions, processing, and cooking [64]. Some degree of misclassification may have occurred in our dietary assessment; however, such misclassification would probably have been more non-differential such that the bias would likely have been toward null.

One of the strengths of this study is the large sample size, allowing us to introduce possible confounders in the models. The use of an established protocol also allowed us to assign the GI values to the SFFQ in a systematic, reproducible manner. Furthermore, to our knowledge, this is the first study conducted among Mexican adolescents to explore the association among dietary GI, dietary GL, and MetS or its components. Nevertheless, further evidence based on prospective studies is necessary to determine the long-term association among dietary GI, dietary GL, and MetS in youth populations.

## Conclusions

We observed no association between dietary GI or dietary GL and MetS in a nationally representative sample of Mexican adolescents. However, we found higher odds of abnormal blood pressure among female adolescents with the highest dietary GI and GL. This investigation contributes to the body of evidence about the relationship between the quality of carbohydrates and MetS risk factors in youth populations. However, owing to the cross-sectional study design, our results have to be treated with caution, and further investigations are required to confirm the identified associations.

## Abbreviations

BMI: Body mass index
CI: Confidence interval
GI: Glycemic index
GL: Glycemic load
HDL-c: High-density lipoprotein cholesterol
IDF: International Diabetes Federation
MetS: Metabolic syndrome
MUFA: Monounsaturated fatty acids
NHNS-2012: National Health and Nutrition Survey 2012
OR: Odds ratio
PUFA: Polyunsaturated fatty acids
RCTs: Randomized controlled trials
SD: Standard deviation
SES: Socioeconomic status
SFA: Saturated fatty acids
SFFQ: Semiquantitative food-frequency questionnaire
T2D: Type 2 diabetes mellitus
WC: Waist circumference
WHO: World Health Organization
